# Monkeypox Virus Transcriptional Profiles and Host Responses in Skin Lesion Swabs Among Individuals With Human Immunodeficiency Virus

**DOI:** 10.1093/infdis/jiaf316

**Published:** 2025-06-11

**Authors:** Jacklyn R Hurst, Darrell H S Tan, Abby Li, Shreya S Khera, Reva Persaud, Adrienne Chan, Sharon Walmsley, Misha Hummel, Cassandra Bertucci, Oscar J Pico Espinosa, Sharmistha Mishra, Russell S Fraser, Robert Kozak

**Affiliations:** Biological Sciences, Sunnybrook Research Institute, Sunnybrook Hospital, Toronto, Ontario, Canada; Division of Infectious Diseases, St Michael's Hospital, Toronto, Ontario, Canada; MAP Centre for Urban Health Solutions, St Michael's Hospital, Toronto, Ontario, Canada; Department of Medicine, University of Toronto, Toronto, Ontario, Canada; Institute of Medical Science, University of Toronto, Toronto, Ontario, Canada; Institute of Health Policy, Management and Evaluation, Dalla Lana School of Public Health, Toronto, Ontario, Canada; MAP Centre for Urban Health Solutions, St Michael's Hospital, Toronto, Ontario, Canada; MAP Centre for Urban Health Solutions, St Michael's Hospital, Toronto, Ontario, Canada; MAP Centre for Urban Health Solutions, St Michael's Hospital, Toronto, Ontario, Canada; Division of Infectious Diseases, Department of Medicine, Sunnybrook Health Sciences Centre, Toronto, Ontario, Canada; Clinical Public Health and Institute of Health Policy, Management and Evaluation, Dalla Lana School of Public Health, University of Toronto, Toronto, Ontario, Canada; Infectious Diseases, Department of Medicine, University Health Network, Toronto, Ontario, Canada; MAP Centre for Urban Health Solutions, St Michael's Hospital, Toronto, Ontario, Canada; MAP Centre for Urban Health Solutions, St Michael's Hospital, Toronto, Ontario, Canada; MAP Centre for Urban Health Solutions, St Michael's Hospital, Toronto, Ontario, Canada; Division of Infectious Diseases, St Michael's Hospital, Toronto, Ontario, Canada; MAP Centre for Urban Health Solutions, St Michael's Hospital, Toronto, Ontario, Canada; Department of Medicine, University of Toronto, Toronto, Ontario, Canada; Institute of Medical Science, University of Toronto, Toronto, Ontario, Canada; Institute of Health Policy, Management and Evaluation, Dalla Lana School of Public Health, Toronto, Ontario, Canada; Department of Pathology and Microbiology, Atlantic Veterinary College, University of Prince Edward Island, Charlottetown, Prince Edward Island, Canada; Biological Sciences, Sunnybrook Research Institute, Sunnybrook Hospital, Toronto, Ontario, Canada; Department of Laboratory Medicine and Pathobiology, University of Toronto, Toronto, Ontario, Canada; Department of Laboratory Medicine and Molecular Diagnostics, Division of Microbiology, Sunnybrook Health Sciences Centre, Toronto, Ontario, Canada

**Keywords:** monkeypox virus, HIV, transcriptomics, host–virus interaction, immune evasion

## Abstract

**Background:**

Mpox disease, caused by the monkeypox virus (MPXV), remains a global health concern with nearly half of all cases occurring among individuals with human immunodeficiency virus (HIV). While recent studies have advanced our understanding of poxvirus pathogenesis, the molecular effects of mpox and HIV coinfection are still poorly understood. This study uses dual RNA sequencing (RNA-seq) to characterize host and viral gene expression in skin lesion swabs from people with mpox, including individuals with and without HIV.

**Methods:**

Our cohort included 19 participants with confirmed MPXV infection, with 53 total skin lesion swabs collected during the early (7–13 days post–symptom onset) and late (15–21 days post–symptom onset) stages of mpox disease. RNA-seq was used to assess both host and MPXV gene expression over time in participants with and without HIV.

**Results:**

HIV-positive participants showed upregulation of MPXV genes involved in immune evasion and viral replication. Conversely, host immune pathways, such as interferon signaling, apoptosis, and chemokine recruitment, were downregulated in participants with HIV. Pathway enrichment analysis revealed dysregulated immune signaling and autophagy, key processes for viral clearance. These findings suggest that HIV-related immunosuppression may enhance MPXV replication and prolong disease.

**Conclusions:**

This study highlights the use of dual RNA-seq in uncovering molecular interactions between host and virus during mpox infections. Our findings offer insights into how HIV coinfection may alter MPXV pathogenesis, with implications for treatment strategies and disease management in immunocompromised populations.

## Teaser

Dual RNA sequencing of mpox skin lesions reveals distinct viral and host gene expression patterns by HIV status, highlighting how differences in immune context—despite viral suppression—may influence monkeypox virus immune evasion strategies and host antiviral responses.

Mpox disease is caused by the monkeypox virus (MPXV), an enveloped double-stranded DNA virus belonging to the *Orthopoxvirus* genus of the Poxviridae family. The ongoing global outbreak has resulted in >100 000 cases, with approximately 30%–50% of cases occurring in people with human immunodeficiency virus (HIV) infection [1–5], depending on the population and setting studied. Coinfection, particularly in people with advanced HIV disease (eg, low CD4 cell counts), is associated with an increased risk for severe mpox disease, hospitalization, and mortality [3, 6–8]. Individuals with CD4 counts <200 cells/μL are particularly vulnerable to severe and disseminated mpox, with complications including prolonged illness, extensive skin lesions (>100), multiorgan involvement, and mortality rates as high as 15%–27% [6, 8–10]. Moreover, recent studies indicate that individuals with a CD4 count <350 cells/μL likely have a reduced ability to mount a cell-mediated immune response against MPXV, which may contribute to disease severity [5]. Collectively, this highlights the need for further investigation into the interplay between mpox and HIV infections and its impact on host pathogenesis.

Important advances have been made in understanding the pathogenesis of poxviruses using host gene expression analyses. However, studies have primarily focused on vaccinia virus (VACV) as a model organism, and expression profiles induced by infection with different orthopoxviruses vary significantly despite their close genetic relationship [11]. While many studies have explored the transcriptional impact of mpox infection across various cell types predominantly utilizing microarray-based techniques [11–14], microarrays are limited to analyzing only those transcripts for which probes or gene-specific primers exist. In contrast, total RNA sequencing (RNA-seq) methods allow for the examination of the entire transcriptome. Specifically, dual RNA-seq offers a powerful and accessible method to explore these differences, enabling comprehensive analysis of host and viral gene expression that permits objective and simultaneous analysis of both host and viral gene profiles. Previous work has shown that dual RNA-seq can uncover complex interactions between viruses and their host cells at the transcriptomic level [15, 16].

Host gene expression during MPXV infection has been extensively performed in mammalian cell culture models, including HeLa cells [17, 18], HaCat keratinocytes [19], and African green monkey cells [20–22] in an effort to elucidate host responses. Recently, host gene expression was characterized in a human cystic skin organoid model and revealed profound rewiring of the host transcriptome during active MPXV infection [23]. However, viral transcriptomic signatures underlying MPXV and its interaction with the host immune system remain largely unexplored. Thus, dual RNA-seq of people with mpox may reveal unique features of MPXV pathogenesis and identify host biomarkers during disease progression. By analyzing gene expression patterns directly from clinical samples, such as skin lesion swabs from people with mpox infection, we can gain a more accurate understanding of these host–virus interactions and the impact of coinfections on viral dynamics and host responses during disease progression. Here, we demonstrate the feasibility of using dual RNA-seq on lesion samples from individuals with mpox infection to simultaneously characterize both host and MPXV gene expression and compare their gene signatures over time by HIV status. Since coinfection with HIV has been associated with prolonged and more severe mpox disease, we compared transcriptional profiles from lesion swabs collected at early (7–13 days post–symptom onset) and late (15–21 days post–symptom onset) stages in HIV-positive and -negative participants. Exploring gene expression in these groups will improve our understanding of how coinfections drive MPXV pathogenesis and could assist in identifying key host response pathways activated or suppressed in the presence of HIV coinfection.

## MATERIALS AND METHODS

### Participant Cohort and Sample Collection

Study participants were recruited between June and October 2022 as part of the Mpox Prospective Observational Cohort Study (MPOCS), an ongoing cohort study of individuals with confirmed MPXV infection that involves systematic longitudinal collection of biological samples [24]. The study was approved by the Research Ethics Board of Unity Health Toronto (CTO ID number 4081, UHT REB number 22-130). All participants provided written informed consent and received financial compensation for participating in the study. Eligibility criteria for biological samples included only those with microbiologically confirmed MPXV infection. Participants underwent a baseline clinical assessment by an infectious disease physician researcher as soon as possible after the diagnosis was suspected by a healthcare provider. The first research visit included an interview and participant questionnaire for characterization of the participant's illness, epidemiologic exposure history, medical history, and demographics. All participants underwent sampling from multiple sites (eg, skin, nasopharynx, throat, rectal, urine). For this study, skin lesion swabs were chosen due to the high viral load and presence of viable virus in lesion samples [25–27]. A minimum of 1 lesion swab was collected from each participant during their visit, with the anatomical location of the lesion varying for each participant. The stage of each lesion varied at the time of the sample collection and included lesions at macules, papules, vesicles, pustules, and crusted lesions. All skin lesion swabs were collected by trained study staff into vials containing viral transport medium. All specimens were refrigerated at 4°C while awaiting transportation to the study storage facility and frozen at −80°C within 24 hours of collection. In-person follow-up visits and continued lesion swab collections were conducted by study staff weekly (±3 days) until 1 week after resolution of all symptoms, where possible.

### RNA Sequencing and Analysis

RNA integrity was assessed using the Agilent 2100 Bioanalyzer. Complementary DNA libraries were prepared with the Illumina Total RNA prep kit following the manufacturer's instructions, including adapter ligation and polymerase chain reaction amplification. Libraries were sequenced as paired-end reads at the Donnelly Sequencing Centre at the University of Toronto (Toronto, Canada). Raw reads underwent metagenomic profiling with the DRAGEN Metagenomics app (v3.5.12) and Kraken2 (v2.0.8 beta) under DRAGEN core v3.5.7 to classify human and MPXV RNA content. For host transcriptome alignment, sequencing data were processed on Illumina Connected Analytics using the DRAGEN RNA pipeline (v.4.2.4). Reads were mapped to the human reference genome (1000 Genomes GRCh38 v3 multigenome; GENCODE Release 29. Separate DRAGEN runs for viral reads were performed using the MPXV reference genome and annotations (National Center for Biotechnology Information [NCBI] NC_063383.1) to assess viral gene expression. Transcript abundance was normalized as transcripts per million, accounting for transcript length and sequencing depth. Differential gene expression analysis was conducted using DESeq2 (v1.26.0), generating log_2_ fold changes and false discovery rate–adjusted *P* values. Visualization of differentially expressed genes (DEGs) in heatmaps and MA plots and sample clustering by principal component analysis (PCA) were all generated in R (for MacOS) software.

### Analysis of Differentially Expressed Viral Genes

For interpretive purposes, viral DEGs were defined as those with an adjusted *P* value (*P*_adj_) <.05 and fold change >1.5. A formal enrichment analysis was not performed for viral genes due to the absence of established pathway enrichment libraries for MPXV. Instead, all significantly differentially expressed MPXV genes were categorized into broad functional groups—viral genome replication and transcription, viral entry, host modulation, and assembly and budding—based on annotations from UniProt, NCBI (NC_063383.1), and published literature describing MPXV gene functions and VACV orthologs [14, 28–30]. MPXV genes for which no functional annotation could be identified or inferred were designated as unknown.

### Analysis of Differentially Expressed Host Genes

The significance of differentially expressed host genes was identified using a fold change >2 combined with *P*_adj_ < .01. To identify enriched biological pathways, Gene Ontology (GO) enrichment for Biological Processes was performed for all statistically significant upregulated and downregulated genes using the clusterProfiler R package. Differential expression results were filtered to remove missing values, and Ensembl gene IDs were cleaned to exclude version numbers. Genes were ranked by log_2_ fold change and analyzed using the enrichGO() function with the org.Hs.eg.db annotation, applying Benjamini–Hochberg correction (*q* < .05). Results were visualized using dot plots of the top enriched terms.

## RESULTS

### Participant Characteristics

We analyzed 53 skin lesion swabs from 19 participants: 31 swabs from 11 HIV-negative participants and 22 swabs from 8 HIV-positive participants. All participants identified as gay men. Among participants with HIV, the median CD4 count was 360 cells/μL (interquartile range [IQR], 224–901 cells/μL), a level associated with reduced cell-mediated immunity to MPXV [6]. Smallpox vaccination history varied: 4 participants received a smallpox vaccine prior to 2022 (3 HIV-positive, 1 HIV-negative) while 5 received the third-generation Modified Vaccinia Ankara–Bavarian Nordic vaccine (Imvamune) in 2022 (2 HIV-positive, 3 HIV-negative). Participant demographics, including HIV indices, vaccination status, and tecovirimat exposure, are detailed in Table 1. To confirm comparability across groups, we examined the time from symptom onset to sample collection and found no significant differences between HIV-positive and HIV-negative participants at either the early or late phase (Supplementary Figure 1).

**Table 1.** Characteristics of Study Participants

Characteristic	HIV-Negative Participants (n = 11)	HIV-Positive Participants (n = 8)
Participants sampled in early disease stage	5 (45)	7 (88)
Participants sampled in late disease stage	11 (100)	5 (63)
Demographics		
Age, y, median (IQR)	35 (32–39)	40 (34–53)
HIV indices		
HIV-negative, on PrEP	7 (64)	…
HIV-negative, not on PrEP	4 (36)	…
Plasma HIV RNA <20 copies/mL	…	Undetectable for all
CD4 count, cells/μL, median (IQR)	Not determined	360 (224–901)
Vaccination status		
Smallpox vaccine prior to 2022	1 (9)	3 (38)
Unsure of smallpox vaccine prior to 2022	3 (27)	0 (0)
Imvamune (MVA-BN) vaccine in 2022	3 (27)	2 (25)
Received 1 dose	3 (27)	2 (25)
Received 2 doses	1 (9)	1 (13)
Doses received as PrEP	2 (18)	2 (25)
Doses received as PEP	0 (0)	1 (13)
Exposure to tecovirimat		
Participants who underwent tecovirimat treatment	3 (27)	3 (38)
Interval between symptom onset and treatment initiation, d, median (range)	18 (14–22)	8 (6–10)
Skin lesion swabs, No. (%) of swabs		
Total No. of lesion swabs assessed	31	22
Genital	4 (13)	0 (0)
Perianal	1 (3)	1 (4)
Face	7 (23)	5 (23)
Hands and feet	7 (23)	5 (23)
Trunk and extremities	12 (39)	11 (50)
Early disease stage lesion swabs	11 (35)	15 (68)
Late disease stage lesion swabs	20 (65)	7 (32)

Data are presented as No. (%) of participants unless otherwise indicated.

Abbreviations: HIV, human immunodeficiency virus; IQR, interquartile range; MVA-BN, Modified Vaccinia Ankara–Bavarian Nordic; PEP, postexposure prophylaxis; PrEP, preexposure prophylaxis.

### Quantification of Host and MPXV Reads

To account for coverage variability, we quantified MPXV and human reads in lesion swabs. Specimens with >1000 mapped MPXV reads were included in the viral expression analyses, while the host differential expression was restricted to samples with >1 million mapped human reads (Supplementary Figure 2). In HIV-negative participants, MPXV reads averaged 6 463 860 reads in early disease and 2 310 278 in late disease. In HIV-positive participants, averages were 4 491 586 MPXV reads in early disease and 282 226 in late disease. Human RNA reads were lower than expected, averaging 5 164 835 in early disease and 8 552 080 in late disease for HIV-negative participants, and 5 157 192 and 2 174 716 in early and late disease, respectively, for HIV-positive participants.

### Viral Gene Expression Between Early and Late Phases of Disease

To characterize temporal changes in MPXV gene expression, we analyzed RNA-seq data from lesion swabs collected during early and late stages of infection in HIV-positive and HIV-negative participants. Although similar numbers of differentially expressed MPXV genes were identified from both HIV-positive and HIV-negative participants (Supplementary Table 1), the transcriptional patterns differed markedly by HIV status.

**Figure 1.**
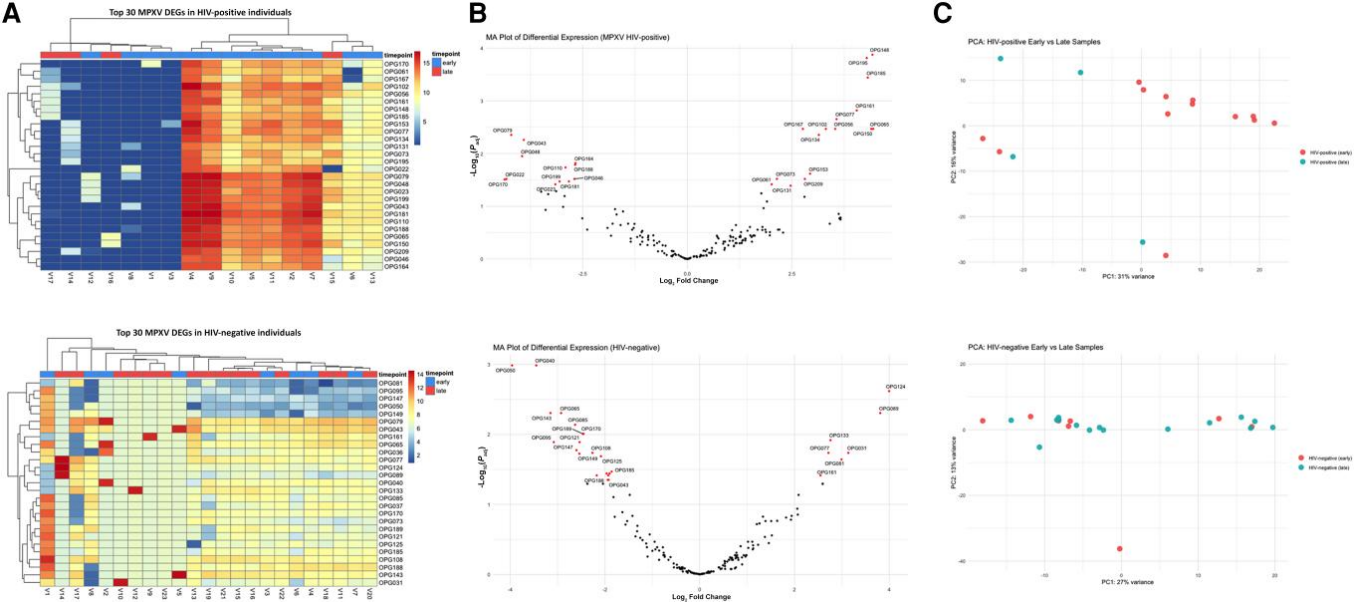
Temporal changes in monkeypox virus (MPXV) gene expression in skin lesion swabs from human immunodeficiency virus (HIV)–positive and HIV-negative participants. MPXV transcriptional profiles were analyzed from lesion swabs collected during early and late stages of mpox disease (7–13 days and 15–21 days post–symptom onset, respectively). *A*, Heatmaps of the top 30 differentially expressed MPXV genes in HIV-positive (top) and HIV-negative (bottom) participants. Rows represent genes and columns represent individual samples, clustered by similarity. Color intensity reflects raw expression values, showing temporal shifts in viral gene expression patterns. *B*, MA plots displaying log_2_ fold changes vs mean normalized expression for MPXV genes in HIV-positive (top) and HIV-negative (bottom) participants. Significantly differentially expressed genes (DEGs) (adjusted *P* < .05) are shown in red with the top 10 most significant genes labeled, highlighting dynamic viral gene regulation over time. *C*, Principal component analysis (PCA) plots showing clustering of early and late lesion swabs by timepoint for HIV-positive (top) and HIV-negative (bottom) participants. Each point represents a sample, colored by timepoint. Clear separation along principal components indicates temporal divergence in MPXV transcriptomes.

Heatmaps of the top 30 differentially expressed MPXV genes (Figure 1*A*) and MA plots (Figure 1*B*) highlight the distinct temporal expression shifts over time. In HIV-positive individuals (Figure 1*A*, top), late-stage lesions showed upregulation of genes involved in immune evasion and viral dissemination, including *OPG195* (inhibits major histocompatibility complex class I [MHC-I] trafficking), *OPG185* (complement evasion), *OPG065* (suppresses antiviral responses), and *OPG161* (promotes cell-to-cell spread). Conversely, genes such as *OPG022*, *OPG170*, and *OPG188*, involved in cytokine binding and interferon (IFN) signaling interference, were downregulated (Figure 1*B*; Supplementary Table 2). In contrast, HIV-negative individuals displayed a different pattern of MPXV gene expression over time (Figure 1*A*, bottom). Notably, *OPG065* was downregulated, and genes such as *OPG143*, *OPG147*, *OPG170*, and *OPG189*—involved in membrane fusion, viral entry, chemokine binding, and extracellular enveloped virus (EEV) release—also showed decreased expression. *OPG031*, an inhibitor of NF-κB signaling, was upregulated in late-stage lesions from HIV-negative individuals. PCA (Figure 1*C*) revealed a clear separation of early and late samples in HIV-positive individuals, indicating consistent temporal transcriptional shifts in viral gene expression. This separation was less pronounced in HIV-negative individuals, suggesting that HIV-related immunosuppression may amplify or shape the dynamics of MPXV gene expression.

**Figure 2.**
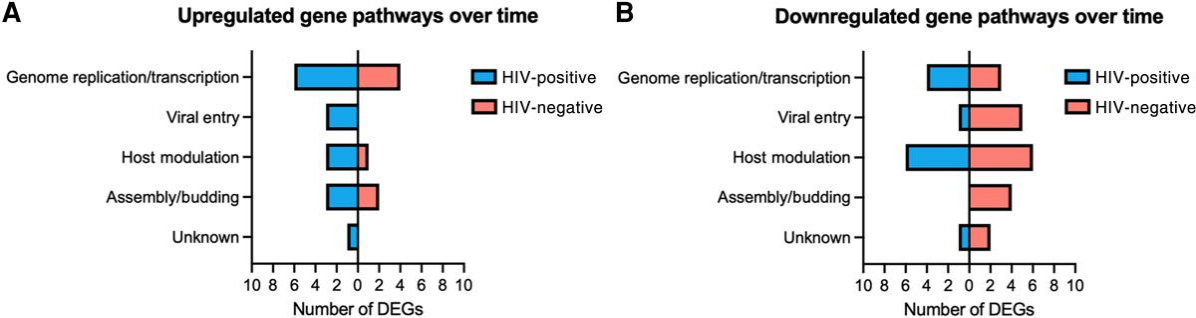
Dysregulated viral biological processes between early and late disease lesion swabs. The functions of all significantly upregulated (*A*) and downregulated (*B*) monkeypox virus (MPXV) genes from skin lesion swabs were identified and compared between human immunodeficiency virus (HIV)–positive and HIV-negative participants. MPXV genes for which no functional annotation could be identified or inferred were designated as unknown. Abbreviations: DEG, differentially expressed gene; HIV, human immunodeficiency virus.

To explore the functional implications of these transcriptional changes, differentially expressed viral genes were annotated by predicted biological function, revealing distinct patterns of viral gene activity between HIV-positive and HIV-negative individuals (Figure 2). Among HIV-positive participants, upregulated genes were primarily involved in genome replication/transcription and immune modulation, whereas HIV-negative individuals showed upregulation of genes associated with viral assembly/budding. In contrast, downregulation in HIV-negative participants was broader and included genes related to viral entry, host modulation, and assembly, suggesting stronger suppression of viral immune evasion mechanisms over time. These patterns suggest that HIV status may shape the trajectory of viral gene expression during lesion progression.

### Dysregulation of Host Genes and Pathways

To explore the impact of MPXV and HIV status on host pathogenesis, we analyzed changes in host gene expression over time in lesion swabs collected during early and late stages of disease. HIV-positive individuals exhibited markedly greater transcriptomic shifts than HIV-negative participants, with 3329 DEGs identified between early and late timepoints (*P*_adj_ < .01; Supplementary Table 1). In contrast, lesions from HIV-negative individuals showed minimal host gene dysregulation over time, with only 2 genes significantly altered.

**Figure 3.**
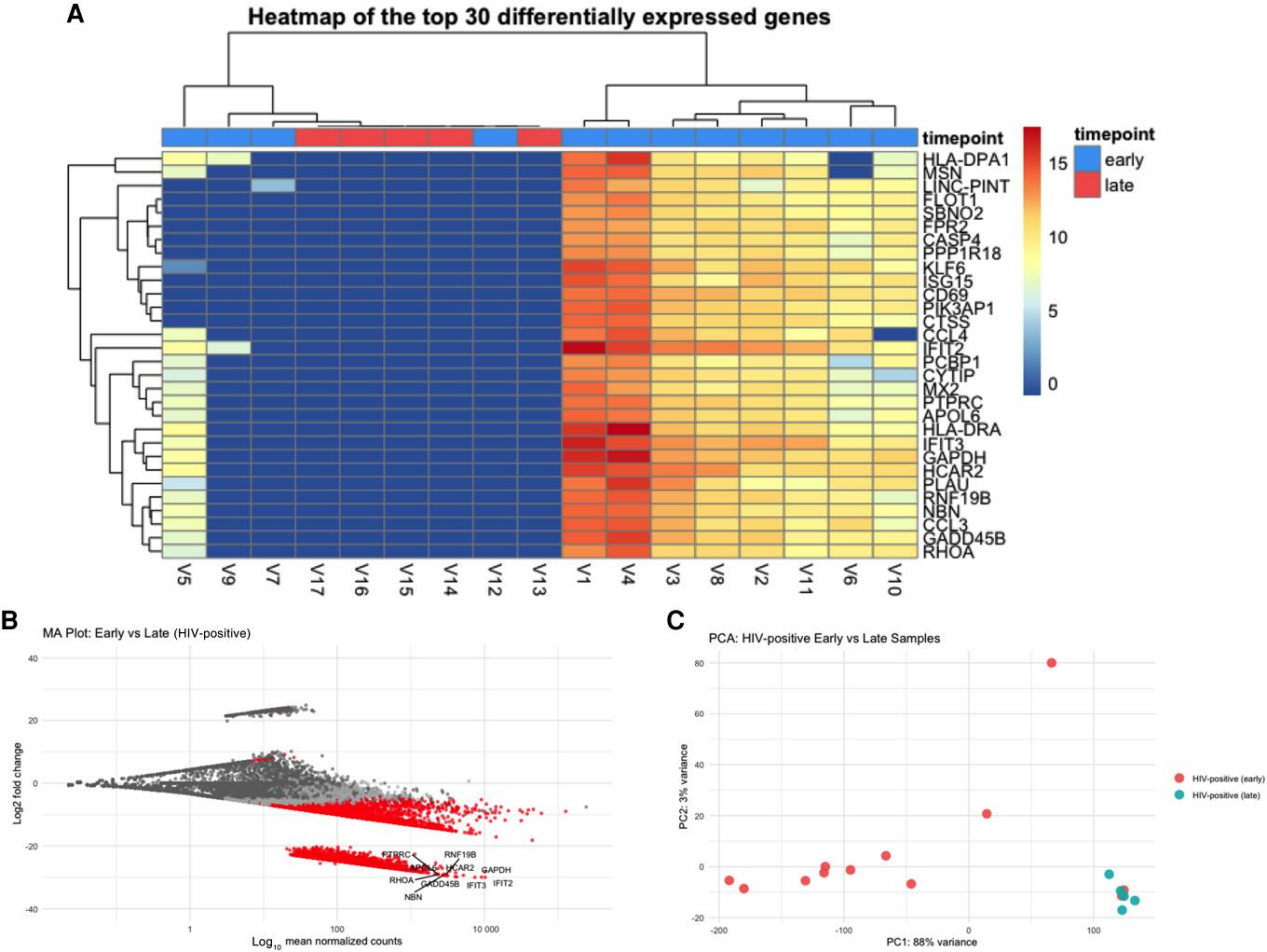
Transcriptomic profiles of lesion swabs from human immunodeficiency virus (HIV)–positive individuals with monkeypox virus infection over time. Skin lesion swabs were collected during early and late stages of mpox disease and host responses were observed through differential gene expression. Each panel illustrates transcriptomic shifts in HIV-positive participants over time between early and late samples. *A*, Heatmap of top 30 differentially expressed genes (DEGs). Raw expression values are shown in the scale bar. The heatmap shows distinct clustering of late disease samples, differing from the majority of early disease samples, suggesting notable changes in host gene expression over the course of the infection. *B*, MA plot displaying log_2_ fold changes vs mean normalized counts. Genes with adjusted *P* values <.01 are considered significantly differentially expressed and are represented as red dots. The top 10 DEGs are labeled, all downregulated. *C*, Principal component analysis (PCA) plot with samples colored by disease stage. Each dot represents an individual lesion swab included in the analysis. Early and late disease samples separate into distinct clusters, highlighting significant transcriptomic shifts over time.

As shown in Figure 3*A*, hierarchical clustering of the top 30 DEGs revealed clear segregation of late-stage samples, indicating substantial remodeling of host gene expression during disease progression in people with HIV. The MA plot (Figure 3*B*) further highlights the predominance of downregulated genes at the late timepoint, including key antiviral effectors such as *ifit3* and *stat1*, and chemokine genes *ccl3*, *ccl4*, and *ccr2*, which are involved in immune cell recruitment. These gene expression changes may contribute to impaired antiviral defense and reduced immune cell trafficking to infection sites. Several apoptosis-related genes, such as *GADD45B* and *HCAR2*, were also downregulated, potentially indicating altered cell death pathways. PCA (Figure 3) revealed distinct clustering by timepoint, reinforcing the presence of widespread transcriptional reprogramming in HIV-positive individuals over time.

**Figure 4.**
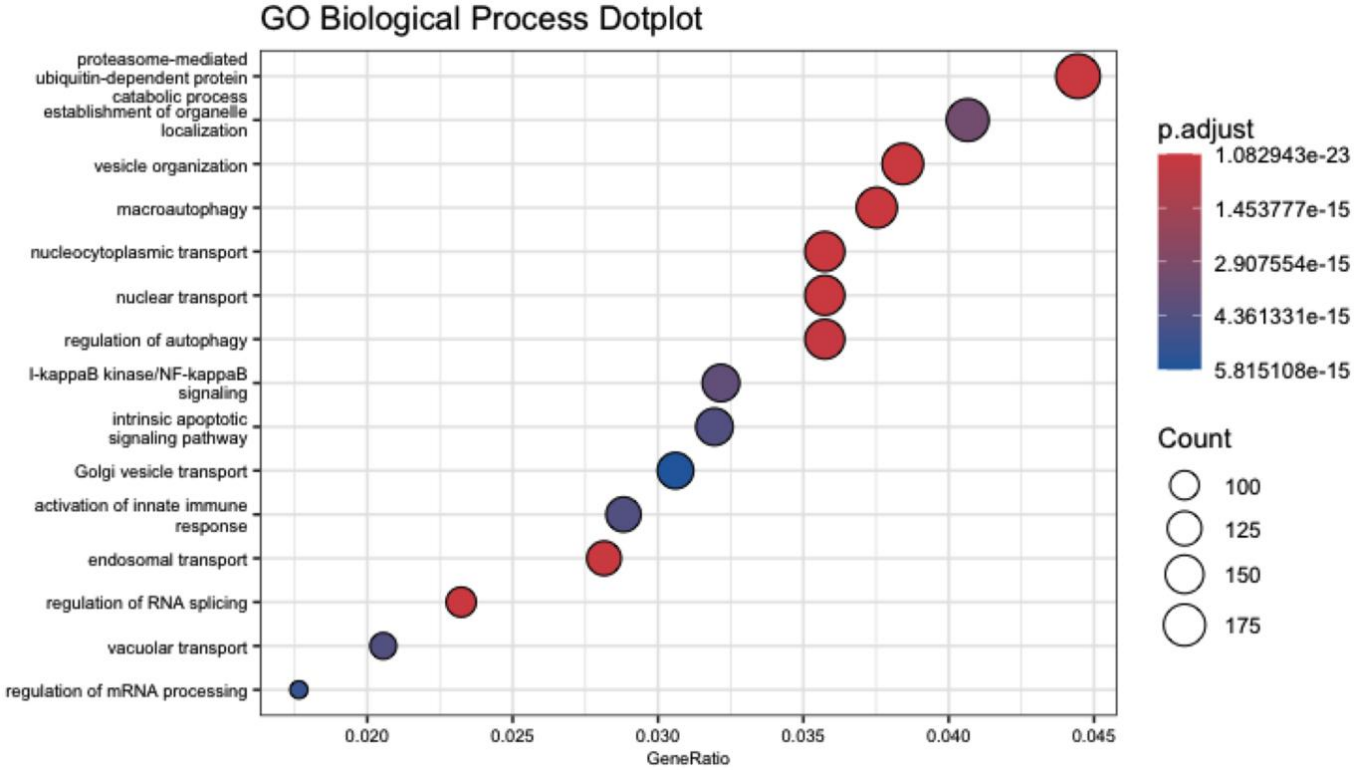
Enriched host pathways between early and late disease lesion swabs in human immunodeficiency virus (HIV)–positive participants. This Gene Ontology (GO) Biological Process dotplot displays the top 15 enriched biological pathways identified from differentially expressed genes (DEGs) between early and late disease lesion swabs. Pathways are ordered from top to bottom based on the GeneRatio shown on the x-axis, which represents the proportion of DEGs in each pathway relative to the total number of genes in that pathway. The size of each dot indicates the number of genes involved in the pathway, while the color of the dot corresponds to the statistical significance of the pathway based on the adjusted *P* values (p.adjust). Red dots represent pathways with lower adjusted *P* values, indicating stronger statistical significance.

To further understand the biological relevance of these gene expression changes, we conducted GO enrichment analysis. The top 15 significantly enriched biological processes (Figure 4) included pathways involved in protein catabolism (eg, proteasome-mediated degradation, ubiquitin-dependent protein catabolic process), vesicle-mediated transport, and nucleocytoplasmic transport. Notably, several pathways linked to autophagy and immune signaling, including the I-κB kinase/NF-κB pathway and activation of innate immune responses, were also enriched. These findings suggest that coinfection with HIV may exacerbate MPXV pathogenesis by suppressing antiviral immune signaling, impairing cellular trafficking, and altering key regulatory processes such as autophagy and apoptosis.

## DISCUSSION

Coinfection of MPXV and HIV is associated with worse clinical outcomes, including more severe mpox disease, prolonged time to healing of lesions, and higher mortality, particularly among those with low CD4 cell counts and high HIV viral load [3, 4, 6–8]. Despite this, no studies have simultaneously examined host and viral gene expression during MPXV and HIV coinfection. Our dual RNA-seq approach addresses this gap by characterizing viral and host transcriptional responses in lesion swabs from individuals with and without HIV. Given the substantial portion of the viral genome dedicated to modulating host responses [29], viral gene expression provides critical insight into pathogenesis.

Early mpox disease is characterized by active lesion formation and high viral shedding, typically within the first 2 weeks of illness, followed by scab formation and declining infectiousness in later stages [6, 25]. We defined early disease as 7–13 days and late disease as 15–21 days post–symptom onset, reflecting the typical onset of lesion resolution around day 14 [6, 25, 27]. No samples were collected on day 14 ensuring a clear—though narrow—separation between phases. While a broader sampling window could improve biological distinction, our cutoffs align with prolonged infectious periods observed in immunocompromised individuals, including those with HIV [25, 27]. Supporting this framework, up to 86% of skin lesion samples collected during the late period (15–21 days post–symptom onset) still test positive for MPXV DNA, regardless of immune status [26, 27, 31]. We compared time from symptom onset to sampling time for both HIV-positive and HIV-negative individuals and found no significant differences between the groups for either early or late sample collection (Supplementary Figure 1), supporting the consistency of our phase definitions and minimizing potential bias. Although future studies with denser sampling are warranted, our framework captures the key transition from peak viral activity to disease resolution.

Among HIV-positive participants, several MPXV genes involved in immune evasion were upregulated between early and late disease. Mpox infection typically activates cytotoxic T cells through MHC-I presentation, triggering cell killing through cytokines like IFN gamma (IFN-γ) and tumor necrosis factor alpha (TNF-α), or through direct lysis [32]. We saw upregulation of *OPG195*, which acts to evade T-cell detection of infected cells by impairing peptide loading and inhibiting MHC-I molecule trafficking in the endoplasmic reticulum [33]. This is a common immune evasion strategy among poxviruses, including cowpox virus (CPV) [34] and Myxoma virus [35], and leads to a dramatic reduction in cytotoxic T-cell activity. Virus-specific CD8^+^ T-cell responses have been shown to be reduced by >90% when stimulated with MPXV compared to VACV and do not trigger inflammatory cytokine production (IFN-γ or TNF-α) by virus-specific T cells [34]. The evasion tactics used by MPXV to counter immune surveillance by virus-specific T cells may explain why MPXV is able to spread efficiently. *OPG065*, which suppresses antiviral responses to double-stranded RNA (dsRNA), was also upregulated, reflecting mechanisms seen in VACV and CPV to evade Toll-like receptor (TLR)–mediated defenses [36–38]. Binding of viral dsRNA to TLRs can activate protein kinase R and trigger the expression of proinflammatory molecules involved in host antiviral responses and subsequent activation of the adaptive immune defense system, including IFN-mediated responses. A transcriptomic analysis of the host response to mpox infection in multiple cells revealed repression of TLR3 target genes in MPXV-infected cells, and transcripts from the predicted dsRNA binding protein were also detected during infection in these cell types [14], supporting our findings. Additionally, *OPG185*, which protects infected cells and EEVs from complement attack, was upregulated. The VACV homologue A56 protein has been shown to bind to other viral proteins and anchor them to the surface of an infected cell, including the vaccinia virus complement control protein that can protect infected cells from complement [39]. Together, the upregulation of MPXV genes involved in immune modulation reflects viral strategies commonly employed by poxviruses to enhance immune evasion [40]. Interestingly, only 1 MPXV immune modulatory gene was upregulated in the non-HIV group over time—*OPG031*, involved in the inhibition of NF-kB activation.

Our host gene expression analysis revealed that several immune-related genes were dysregulated between early and late stages of mpox disease in lesions from people coinfected with HIV, a pattern that was absent in lesions from people with mpox infection only. Downregulation of innate immune genes (*ifit3*, *stat1*) and chemokine-related genes (*ccl3*, *ccl4*, *ccr2*) suggests impaired antiviral defenses and lymphocyte recruitment. Given the central role of *ifit3* and *stat1* in IFN signaling pathways, their downregulation suggests that HIV coinfection may compromise the host's ability to mount an effective IFN response, which could facilitate MPXV replication and persistence in people with HIV. These findings align with previous reports of dysregulated chemokine and IFN responses in people living with HIV that increase susceptibility to opportunistic infections [41, 42]. The downregulation of *ccl3* and *ccl4*, which are crucial for recruiting immune cells, along with *ccr2*, which mediates monocyte trafficking, suggests impaired immune cell recruitment to lesions. Downregulation of apoptosis-related genes *GADD45B* and *HCAR2* further suggests that MPXV may interfere with apoptotic pathways to enhance host cell survival and viral replication [33]. Together, an impaired immune response over time in people with HIV could contribute to prolonged viral shedding and delayed lesion healing and may explain the increased severity of MPXV infection in this population.

Pathway enrichment analyses in HIV-positive participants identified dysregulation in ubiquitin-mediated processes, autophagy, and innate immune signaling. Ubiquitination is central to innate immune responses, facilitating the degradation of viral components and their presentation to immune cells. MPXV exploits host ubiquitin and autophagy systems to enhance replication and evade detection [33, 43], which may be intensified by HIV-induced immunosuppression. Similar disruptions have been observed in HIV coinfection with herpesvirus and hepatitis C virus [44–46], including downregulation of genes involved in cytokine signaling and IFN-mediated responses [47]. These findings suggest shared dynamics in HIV coinfections, where immunosuppression modifies viral evasion strategies. Although our participants had undetectable HIV viral loads and none had low CD4 T-cell counts, future studies in individuals with advanced HIV will further clarify these effects.

This study offers new insights into how HIV coinfection may influence host and MPXV gene expression; however, several limitations must be acknowledged. Some participants were vaccination and/or treated with tecovirimat, which may affect transcriptional responses, though sample size limited subgroup comparisons. Timing of sample collection relative to treatment initiation also varied, potentially influencing observed gene expression patterns. Further, while our threshold for sample inclusion with >1000 MPXV reads and >1 million human reads, ensured high-quality data, this approach may bias results toward samples with higher viral loads and stronger immune responses, possibly overlooking subtle gene expression changes in less severe disease or later-stage infections. Samples with low MPXV reads may still offer valuable information about viral persistence or suppression, and thus, their exclusion may narrow the scope of insights into the full spectrum of viral dynamics in skin lesions. Since the presence of any MPXV mRNA suggests ongoing replication, future studies should include low-read samples to broaden understanding of transcriptional dynamics.

In conclusion, our study sheds light on the complex interplay of mpox infection in people with HIV, revealing significant alterations in both host and viral gene expression. HIV coinfection appears to influence MPXV gene expression, with notable changes in the virus's immune evasion strategies and corresponding changes in host immune responses. Specifically, the upregulation of MPXV genes associated with immune modulation, alongside the downregulation of host genes involved in cytokine signaling and inflammation, suggests that MPXV adapts to exploit the immunocompromised environment created by HIV. These findings highlight the importance of understanding MPXV pathogenesis in the context of HIV coinfection to inform therapeutic strategies and improve patient outcomes.

## Supplementary Material

jiaf316_Supplementary_Data

## Supplementary Data

Supplementary materials are available at *The Journal of Infectious Diseases* online (http://jid.oxfordjournals.org/). Supplementary materials consist of data provided by the author that are published to benefit the reader. The posted materials are not copyedited. The contents of all supplementary data are the sole responsibility of the authors. Questions or messages regarding errors should be addressed to the author.

## Notes

***Author contributions.*** J. R. H. and R. K. conceptualized the study. J. R. H. processed samples, completed the data analysis, and generated all figures and tables. R. P., S. M., S. W., M. H., C. B., O. J. P. E., and A. C. were involved in patient recruitment and sample collection. S. S. K. and A. L. were involved in sample storage and data collection. J. R. H., R. S. F., and R. K. contributed to data interpretation and the initial manuscript draft. All authors contributed to manuscript review and edits. J. R. H., D. H. S. T., S. M., and R. K. all read and approved the final version for submission.

***Data availability.*** Data are available in the Supplementary Material.

***Financial support.*** This work was funded by the Canadian Institutes of Health Research Rapid Mpox Research (grant number MRR-184812). D. H. S. T. is supported by a Tier 2 Canada Research Chair in Biomedical HIV/STI Prevention. S. M. is supported by a Tier 2 Canada Research Chair in Mathematical Modeling and Program Science.
