# Supplementary material for: Monkeypox Virus Transcriptional Profiles and Host Responses in Skin Lesion Swabs Among Individuals With Human Immunodeficiency Virus
Source: J Infect Dis. 2025 Jun 11;232(5):e849–58. doi: 10.1093/infdis/jiaf316 (PMC12614975; doi:10.1093/infdis/jiaf316)
Supplement: jiaf316_Supplementary_Data [file jiaf316_supplementary_data.docx]

**
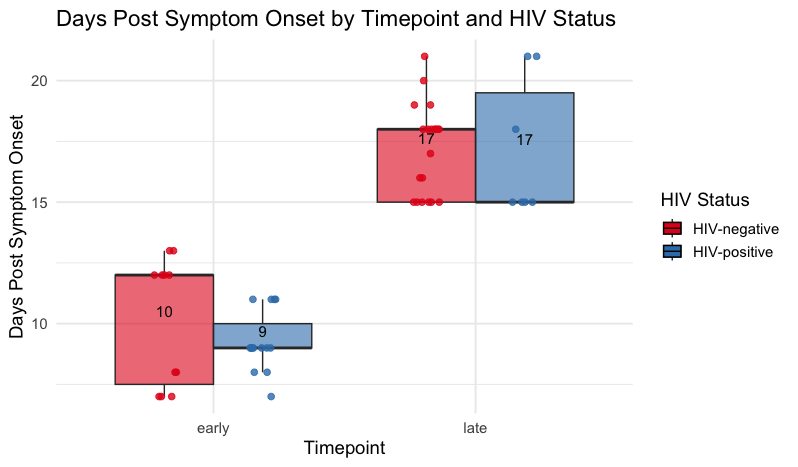
**

**Fig S1. Time from symptom onset to lesion swab collection in HIV-positive and HIV-negative participants.** The range and mean number of days from symptom onset to skin lesion swab collection during early and late stages of mpox infection, stratified by HIV status. Each point represents an individual swab. The box represents the interquartile range with the lower and upper bounds of the box representing the first and third quartiles, and whiskers extending to the minimum and maximum values. Black numbers within each box denote the rounded group means. No significant differences were observed between HIV status groups at either timepoint.


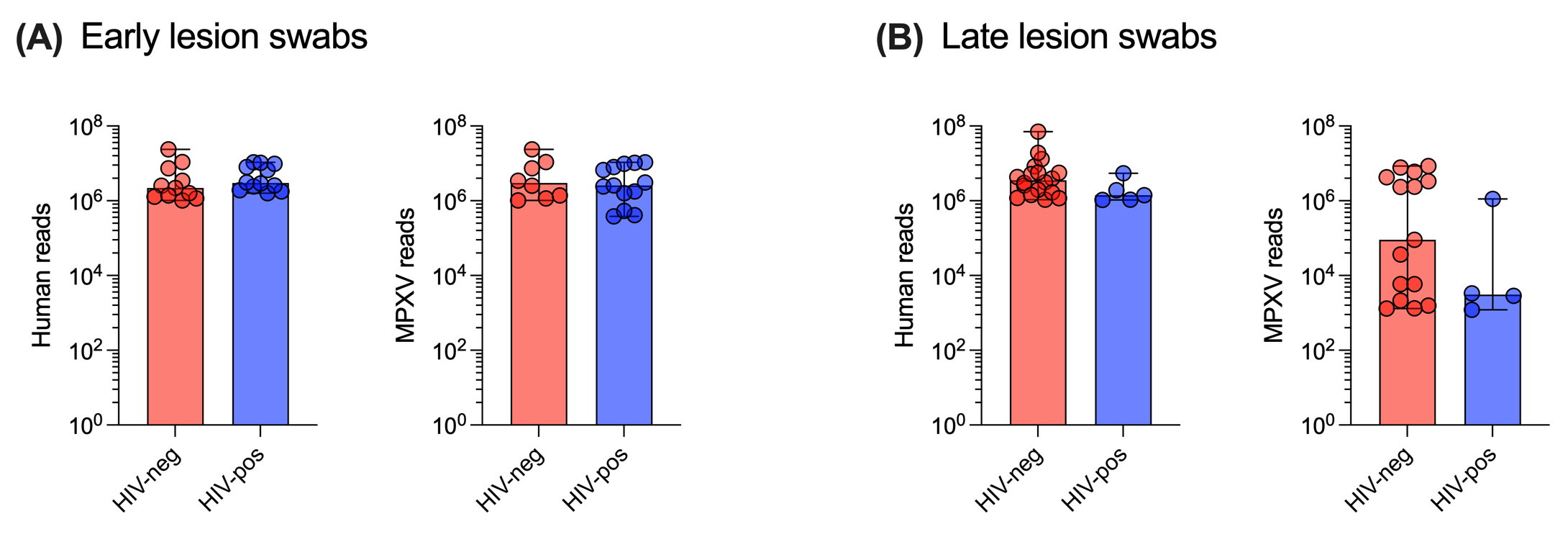
 **Fig S2. Host and virus RNA read counts from mpox skin lesion swabs during early and late disease.** The number of human and MPXV read counts in skin lesion swabs were determined in HIV-positive (HIV-pos) and HIV-negative (HIV-neg) samples collected during **(A)** early and **(B)** late disease. All samples included in the host differential gene expression analyses contained greater than 1 million mapped human read counts. All samples included in the viral differential gene expression analyses contained greater than 1000 mapped MPXV read counts.

**Table S1. Number of skin lesion swab specimen used for differential gene expression analyses**

| Species of gene expression and HIV status of comparison | Number of participants | | Number of skin lesion specimens | | Number of  DEGs | |
| --- | --- | --- | --- | --- | --- | --- |
|  | Early disease | Late  disease | Early  disease | Late  disease | Up | Down |
| MPXV transcriptomics |  | |  | |  | |
| HIV-positive | 7 | 3 | 13 | 4 | 16 | 12 |
| HIV-negative | 3 | 9 | 8 | 15 | 7 | 20 |
| Host transcriptomics |  | |  | |  | |
| HIV-positive | 5 | 5 | 12 | 5 | 14 | 3329 |
| HIV-negative | 5 | 10 | 11 | 18 | 2 | 0 |

Abbreviations: DEGs, differentially expressed genes

**Table S2. Top 10 differentially expressed MPXV genes in lesion swabs from individuals with HIV co-infection and individuals infected only with mpox**

| Group | Expression | Gene | Function | log2Fold-Change | *p-*value | VACV-Cop | VACV-WR | CPX-Bri |
| --- | --- | --- | --- | --- | --- | --- | --- | --- |
| **HIV-negative** | Up | OPG124 | mRNA Capping Enzyme for Late Gene Products | 3.998 | 2.40E-03 | 1 | 1 | 1 |
|  |  | OPG089 | DNA Nuclease for Viral DNA Damage and Repair | 3.810 | 4.96E-03 | 1 | 1 | 1 |
|  |  | OPG133 | Early transcription factor | 2.760 | 1.21E-02 | 1 | 1 | 1 |
|  |  | OPG031 | Inhibition of host NF-kB activation | 3.137 | 1.83E-02 | 1 | 1 | 1 |
|  |  | OPG077 | Telomere-binding protein required for MV production | 2.717 | 1.83E-02 | 1 | 1 | 1 |
|  |  | OPG081 | Envelope protein of the MV | 2.992 | 2.28E-02 | 1 | 1 | 1 |
|  |  | OPG161 | Membrane protein of the enveloped virion involved in cell-to-cell spread | 2.549 | 3.85E-02 | 1 | 1 | 1 |
|  | Down | OPG040 | Superinfection exclusion protein | -3.450 | 1.03E-03 | 1 | 1 | 1 |
|  |  | OPG050 | Early Protein | -3.960 | 1.03E-03 | 1 | 1 | 1 |
|  |  | OPG065 | Inhibits PKR activation, Inhibits IFN-mediated Responses | -2.926 | 4.96E-03 | 1 | 1 | 1 |
|  |  | OPG143 | Myristylated Protein for Viral Entry | -3.151 | 4.96E-03 | 1 | 1 | 1 |
|  |  | OPG085 | Metalloendopeptidase | -2.631 | 7.28E-03 | 1 | 1 | 1 |
|  |  | OPG170 | Chemokine binding protein | -2.451 | 9.81E-03 | 1 | 1 | 1 |
|  |  | OPG189 | EEV Envelopment and Release | -2.465 | 9.81E-03 | 1 | 1 | 1 |
|  |  | OPG121 | Initiate transcription from late gene promoters | -2.535 | 1.29E-02 | 1 | 1 | 1 |
|  |  | OPG095 | IMV surface membrane protein | -3.080 | 1.29E-02 | 1 | 1 | 1 |
|  |  | OPG147 | Entry-fusion complex protein | -2.602 | 1.68E-02 | 1 | 1 | 1 |
| **HIV-positive** | Up | OPG148 | DNA polymerase processivity factor | 4.424 | 1.33E-04 | 1 | 1 | 1 |
|  |  | OPG195 | Inhibitor of MHC class 1 Molecule Trafficking | 4.287 | 1.54E-04 | X | X | 1 |
|  |  | OPG185 | Protects Infected Cells and EEVs from Complement Attack | 4.312 | 3.61E-04 | F | F | 1 |
|  |  | OPG161 | Envelope glycoprotein involved in cell-to-cell spread | 4.049 | 1.51E-03 | 1 | 1 | 1 |
|  |  | OPG077 | Virosomal protein essential for virus multiplication | 3.567 | 2.22E-03 | 1 | 1 | 1 |
|  |  | OPG065 | Inhibits PKR activation, Inhibits IFN-mediated Responses | 4.442 | 3.40E-03 | 1 | 1 | 1 |
|  |  | OPG150 | Initiate transcription from intermediate gene promoters | 4.396 | 3.40E-03 | 1 | 1 | 1 |
|  |  | OPG056 | Promotes IEV Trafficking through Microtubules | 3.5334 | 3.40E-03 | 1 | 1 | 1 |
|  |  | OPG102 | Positive regulation of the poly(A) polymerase and transcription elongation | 3.310 | 3.40E-03 | 1 | 1 | 1 |
|  |  | OPG167 | Positive regulation of cell-cell adhesion, phagocytosis and inflammatory responses | 2.762 | 3.40E-03 | 1 | 1 | 1 |
|  | Down | OPG079 | ssDNA Binding Protein for Cytoplasmic Virus Factories | -4.209 | 4.42E-03 | 1 | 1 | 1 |
|  |  | OPG043 | Monoglyceride Lipase Homolog | -3.908 | 5.49E-03 | D | D | 1 |
|  |  | OPG048 | Provides the Precursors Necessary for Viral DNA Synthesis | -3.948 | 1.12E-02 | 1 | 1 | 1 |
|  |  | OPG164 | Intracellular Transport and Egress of Virions | -2.673 | 1.52E-02 | 1 | 1 | 1 |
|  |  | OPG188 | Nuclease involved in viral evasion of host cGAS-STING innate immunity | -2.681 | 1.63E-02 | F | F | 1 |
|  |  | OPG110 | Late Transcription Elongation Factor | -2.911 | 1.83E-02 | 1 | 1 | 1 |
|  |  | OPG046 | dUTPase involved in nucleotide metabolism | -2.698 | 3.02E-02 | 1 | 1 | 1 |
|  |  | OPG022 | IL-18 binding protein | -4.323 | 3.02E-02 | X | 1 | 1 |
|  |  | OPG170 | Chemokine binding protein | -4.368 | 3.10E-02 | 1 | 1 | 1 |
|  |  | OPG181 | Binds and stabilizes microtubules | -2.830 | 3.37E-02 | 1 | 1 | 1 |

Abbreviations: 1, Gene present; F, Frameshift; D, large deletion(s) [50+ bp or > 1/3 reference length]; X, ORF sequence can’t be identified

**Table S3. Top 10 differentially expressed human genes in lesion swabs from HIV-negative and HIV-positive participants**

| Group | Expression | Gene | Function | log2Fold-Change | *p-*value |
| --- | --- | --- | --- | --- | --- |
| **HIV-negative** | Up | SHANK2 | SH3 and multiple ankyrin repeat domains 2, involved in neurotransmission | 25.102 | 1.33E-22 |
|  |  | DDX20 | ATP-dependent RNA helicase, splices cellular pre-mRNAs | 25.527 | 4.71E-17 |
|  |  |  |  |  |  |
| **HIV-positive** | Up | HYDIN | Axonemal central apparatus assembly, required for ciliary motility | 24.659 | 9.75E-14 |
|  |  | OTX1 | Development of the brain and the sense organs | 24.267 | 2.43E-13 |
|  |  | LRIG3 | Otolith morphogenesis | 24.119 | 3.36E-13 |
|  |  | WDR87 | Testis Development | 23.818 | 6.35E-13 |
|  |  | GABRA2 | Regulation of postsynaptic membrane potential | 23.798 | 6.58E-13 |
|  |  | BMPER | Blood vessel development | 23.798 | 6.62E-13 |
|  |  | NWD1 | Negative regulation of NF-kappaB transcription factor activity | 23.783 | 6.83E-13 |
|  |  | LINC01132 | Long intergenic non-protein coding RNA 1132 | 23.660 | 8.61E-13 |
|  |  | GRID2 | Synaptic transmission, glutamatergic | 23.293 | 1.77E-12 |
|  |  | GABRA4 | Chloride transmembrane transport | 23.257 | 1.90E-12 |
|  | Down | PELI1 | Positive regulation of TLR3 and TLR4, negative regulation of T cell proliferation | -29.044 | 3.80E-28 |
|  |  | HCAR2 | Mediates nicotinic acid-induced apoptosis in mature neutrophils | -29.081 | 3.80E-28 |
|  |  | GADD45B | Positive regulation of apoptosis | -29.366 | 3.80E-28 |
|  |  | CCL3 | MIP-1alpha, recruits and activates polymorphonuclear leukocytes | -29.288 | 4.44E-28 |
|  |  | RNF19B | Involved in natural killer cell and T-cell cytolytic activity | -29.155 | 5.22E-28 |
|  |  | STAT1 | Cell surface receptor signaling pathway via JAK-STAT | -29.261 | 5.22E-28 |
|  |  | APOL6 | Lipid transport | -28.850 | 1.72E-27 |
|  |  | KLF6 | Transcriptional activator involved in B cell development | -29.210 | 1.72E-27 |
|  |  | IFIT3 | Interferon-induced antiviral protein | -29.195 | 1.98E-27 |
|  |  | RMRP | RNA component of mitochondrial RNA processing endoribonuclease | -28.392 | 3.04E-27 |
